# Neuron-to-glia and glia-to-glia signaling directs critical period experience-dependent synapse pruning 

**DOI:** 10.3389/fcell.2025.1540052

**Published:** 2025-02-18

**Authors:** Nichalas Nelson, Vanessa Miller, Kendal Broadie

**Affiliations:** ^1^ Department of Biological Sciences, Vanderbilt University and Medical Center, Nashville, TN, United States; ^2^ Department of Cell and Developmental Biology, Vanderbilt University and Medical Center, Nashville, TN, United States; ^3^ Department of Pharmacology, Vanderbilt University and Medical Center, Nashville, TN, United States; ^4^ Kennedy Center for Research on Human Development, Vanderbilt University and Medical Center, Nashville, TN, United States; ^5^ Vanderbilt Brain Institute, Vanderbilt University and Medical Center, Nashville, TN, United States

**Keywords:** serotonin, insulin, phosphatidylserine, Draper/MEGF-10, *Drosophila*

## Abstract

Experience-dependent glial synapse pruning plays a pivotal role in sculpting brain circuit connectivity during early-life critical periods of development. Recent advances suggest a layered cascade of intercellular communication between neurons and glial phagocytes orchestrates this precise, targeted synapse elimination. We focus here on studies from the powerful *Drosophila* forward genetic model, with reference to complementary findings from mouse work. We present both neuron-to-glia and glia-to-glia intercellular signaling pathways directing experience-dependent glial synapse pruning. We discuss a putative hierarchy of secreted long-distance cues and cell surface short-distance cues that act to sequentially orchestrate glia activation, infiltration, target recognition, engulfment, and then phagocytosis for synapse pruning. Ligand-receptor partners mediating these stages in different contexts are discussed from recent *Drosophila* and mouse studies. Signaling cues include phospholipids, small neurotransmitters, insulin-like peptides, and proteins. Conserved receptors for these ligands are discussed, together with mechanisms where the receptor identity remains unknown. Potential mechanisms are proposed for the tight temporal-restriction of heightened experience-dependent glial synapse elimination during early-life critical periods, as well as potential means to re-open such plasticity at maturity.

## Introduction

Nearly half of all synapses formed during development are selectively eliminated; a pruning process critical for the proper refinement and optimization of neural circuits (Riccomagno and Kolodkin, 2015; Südhof, 2018). Synapse elimination can occur from neuronal cell-intrinsic absorption via ubiquitin-proteasome system (UPS) activation and lysosome-dependent autophagocytosis (Watts et al., 2003; Kuo et al., 2005; Birdsall and Waites, 2019). More recently, however, glial phagocytes have been established to play a central role in synapse pruning (Schafer and Stevens, 2013; Faust et al., 2021; Nelson et al., 2024b). Mammalian glia initially named based on appearance include astrocytes and microglia, which both function as synaptic phagocytes. Microglia are recruited to sites of synapse elimination, whereas astrocytes can be more intimately resident in tripartite synapses (Sofroniew and Vinters, 2009; Wu et al., 2015) *Drosophila* glia first named based on brain location include cortex, ensheathing, and astrocyte-like glia, which can all function as phagocytes. Cortex glia surround neuron cell bodies (Kremer et al., 2017), ensheathing glia surround synaptic neuropils (Freeman, 2015), and astrocyte-like glia are more closely associated with synapses (Markey et al., 2023). Synaptic pruning involves direct phagocytosis of neuronal synapses (Schafer and Stevens, 2013; Wilton et al., 2019), as well as engulfment of extracellular matrix (ECM) maintaining synapse stability (Nguyen et al., 2020; Crapser et al., 2021). Our focus here is on neuron-to-glia signals and glia-to-glia signals directing experience-dependent glial synapse pruning, a process by which glial cells selectively eliminate unnecessary synapses. However, emerging understanding is guided by more general mechanisms of glial phagocytosis.

Glial phagocytes act on many levels to remove entire damaged/apoptotic neurons (MacDonald et al., 2006; Vita et al., 2021), whole axonal or dendritic branches (Yu and Schuldiner, 2014; Riccomagno and Kolodkin, 2015), and single targeted synapses (Fuentes-Medel et al., 2009). *Drosophila* glial phagocytosis has been well studied in the removal of necrotic axons following injury (Doherty et al., 2009; Purice et al., 2017), and the clearance of apoptotic neurons during normal early brain development (Tasdemir-Yilmaz and Freeman, 2014; Vita et al., 2021). In mice but not *Drosophila*, glial phagocytosis to eliminate targeted synapses in mature brain plasticity has also been well studied (Kim et al., 2016; Dzyubenko and Hermann, 2023). We will consider intercellular signaling mechanisms from these diverse glia fields as they inform experience-dependent synapse pruning during critical periods. These juvenile brain developmental windows are characterized by high levels of connectivity remodeling driven by early sensory input to optimize circuits to a variable environment (Dehorter and Del Pino, 2020). The extent of critical period synapse remodeling is greatly elevated compared to adult plasticity (Pizzorusso et al., 2002; Bradshaw et al., 2018). This early-life remodeling in juvenile brains is experiential dose-dependent, temporally-restricted, and normally reversible only until critical period closure, at which point changes become permanent (Dehorter and Del Pino, 2020; Reha et al., 2020). Disruption of this critical period experience-dependent synapse remodeling contributes to a spectrum of developmental neurological disorders (Chung et al., 2015b; Sellgren et al., 2019). Our focus here is on intercellular signals directing experience-dependent glial synapse pruning during critical periods.

Many sensory systems have been used to explore critical period synapse pruning, but few have examined glial mechanisms until recently. Classic mammalian visual system synaptic ocular dominance demonstrated by experience deprivation first defined the field (Hubel and Wiesel, 1970; Hubel et al., 1977). In the whisker somatosensory system, experience deprivation likewise drives synapse pruning and brain circuit remodeling (Erzurumlu and Gaspar, 2012). The *Drosophila* genetic model has more recently defined critical periods to help reveal molecular mechanisms. The larval motor circuit has one such critical period (Awasaki et al., 2006; Ackerman et al., 2021; Coulson et al., 2022). The mushroom body learning and memory center in the juvenile *Drosophila* brain also exhibits a critical period, in which experience-dependent Fragile X Messenger Ribonucleoprotein (FMRP) function is required for appropriate synapse remodeling (Doll and Broadie, 2015; Broadie, 2016). Upstream, olfactory sensory neurons (OSNs) likewise manifest experience-dependent critical period brain synapse remodeling (Sachse et al., 2007; Das et al., 2011; Chodankar et al., 2020). For example, we have focused on ethyl butyrate (EB) odorant-responsive Or42a neuron innervation of the brain antennal lobe VM7 glomerulus, which has an EB dose-dependent, temporally-restricted, and transiently-reversible critical period (Golovin et al., 2019; 2021). Synapse elimination is mediated by identified glia phagocytes during a very short olfactory critical period (Baumann et al., 2024; Miller and Broadie, 2024; Nelson et al., 2024a; Nelson et al., 2024b). This new model opens an avenue to study neuron-to-glia and glia-to-glia signals directing experience-dependent glial synapse pruning during a well-defined critical period.

In this review, we compare glial phagocytosis mechanisms in early development, injury models, and adult plasticity, but focus on the experience-dependent critical period. More specifically, we focus on recent advances from the *Drosophila* olfactory critical period. We highlight parallels to whole-neuron glial phagocytosis in *Drosophila* juvenile brains, but focus on intercellular signaling for experience-targeted synapse elimination. We attempt to broaden consideration of this pruning cascade beyond singular “find me”, “eat me”, and “don’t eat me” signals, to expand a layered decision-making hierarchy with a more comprehensive consideration of the stages culminating in glial phagocytosis. This expansion includes signals for glial activation, glial-glial signaling, and steps leading to the specific pruning of individual synapses. We attempt to provide a comprehensive list of known signaling ligand/receptor pairs directing glial phagocytosis, with the glial classes involved, life stage implicated, and proposed roles. Both mammalian and *Drosophila* studies are compiled for this comparison, but we pay particular attention to recent advances from new *Drosophila* olfactory critical period work. We end by discussing open questions about glial intercellular communication, considering future studies needed on temporal-restriction mechanisms, and posing the timescale conundrum of experience input driving glial activation/infiltration versus later glial phagocytosis. We also discuss the spatiotemporal retention of signaling from diffusible gradients to localizing small membrane molecules, and the mechanisms by which individual synapses are tagged for removal, and then destabilized/internalized to accomplish synapse elimination.

## Hierarchy of glial pruning stages

**FIGURE 1:**
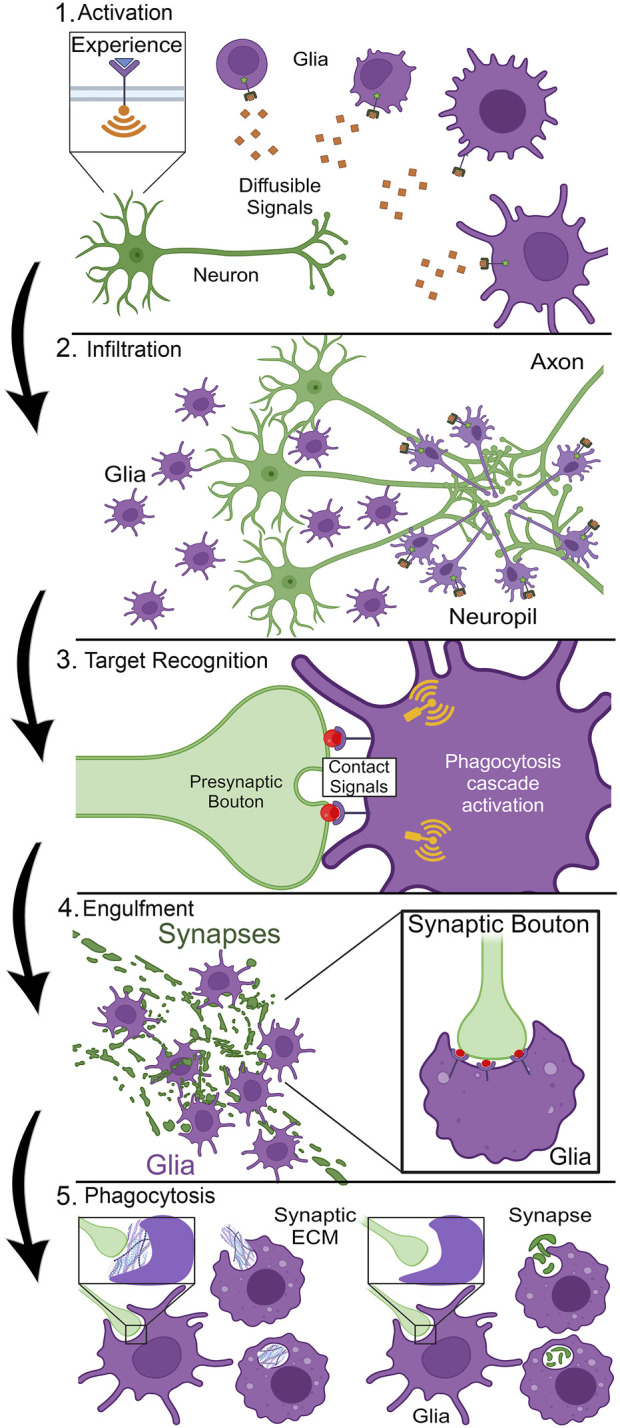
Stages of critical period experience-dependent glial synapse elimination. Stage 1, Activation: Sensory experience triggers neuronal activity to drive the secretion of long-distance diffusible signals that cause glial activation with cellular morphogenesis. Stage 2, Infiltration: Guided by signal gradients acting like morphogens, glia migrate or extend membrane projections into the neuropil to the designated site of synapse pruning. Stage 3, Target Recognition: Cell surface contact signals label the target synapse for glial recognition via cell surface receptors, initiating phagocytosis cascade signaling in the glia. Stage 4, Engulfment: Glia undergo morphological changes based on actin cytoskeleton dynamics to surround the synapse via the formation of a phagocytic cup (see inset, right). Stage 5, Phagocytosis: Glia ingest synaptic material for digestion within phagolysosomes; either foundational synaptic ECM (“synaptomatrix,” left) or part/all of the synapse (right).

Glia have well-established roles pruning brain circuits ranging from whole neurons down to single targeted synapses (Paolicelli et al., 2011; Yu, 2022), but the question is: how is this highly complex pruning process orchestrated? Phagocytosis must be precisely controlled, given the obvious dangers of promiscuous glial pruning. The first step in the cascade is glial activation (Figure 1). Mammalian astrocyte activation involves elevated glial fibrillary acidic protein (GFAP) expression and expansion of the cytoskeleton in the reactive glial cells (Escartin et al., 2021; Edison, 2024). Microglia exhibit a highly ramified architecture (Davalos et al., 2005; Nimmerjahn et al., 2005), with highly motile processes presumably searching for neuronal phagocytosis targets (Vidal-Itriago et al., 2022). Glial activation results in very striking changes in cellular morphology (Figure 1), a phenomenon well-characterized in both astrocytes and microglia (Cornell et al., 2021; Escartin et al., 2021). In *Drosophila*, glial activation is less well characterized. In injury models following axonal severance, ensheathing glia expand their membrane area (MacDonald et al., 2006; Doherty et al., 2009), like microglia shifting from a ramified to amoeboid-like state, even if such characterization fails to capture the diversity of the morphological activation states (Vidal-Itriago et al., 2022). Presumably, long-distance diffusible cue(s) signal via glial receptors to instruct activation (Figure 1). It is not clear how many signals may be involved in glial activation, or indeed whether this cell-state transition represents one step or a series of transitions. Thus, glial activation is a poorly described priming stage in which the molecular players (ligands and receptors) remain largely unknown.

Once activated, whole glia or just their infiltrating processes (Zhao et al., 2015; Butler et al., 2021), need to mobilize to the site of phagocytosis (Figure 1). This infiltration mechanism presents numerous logistical challenges. The brain neuropil is a complex, dense, compacted environment, which presumably acts as a barrier to glial infiltration (McRae and Porter, 2012), including a poorly-defined thicket of extracellular matrix (ECM; Crapser et al., 2020). Glia are suggested to secrete ECM-degrading enzymes, such as matrix metalloproteinases (MMPs), to facilitate neuropil infiltration (Purice et al., 2017; Crapser et al., 2021). In mammals, elevated secreted MMP-9 production is sufficient to induce synaptic remodeling (Huntley, 2012). Likewise, disruption of astrocytic MMP-9 results in precocious closure of the mouse visual critical period, indicating glial dissolution of the ECM enables remodeling (Ribot et al., 2021). In *Drosophila*, neural injury results in glial transcriptional upregulation of secreted MMP-1, which facilitates severed axon clearance (Purice et al., 2017). To direct infiltration, glia presumably navigate via diffusible signal concentration gradients, acting similarly to morphogens. Following such signals, glia must rearrange their actin cytoskeleton in a controlled and directed manner to enable infiltration to the site of phagocytosis (Sepp and Auld, 2003; Marmor-Kollet et al., 2023; Nelson et al., 2024b). It is not known how glia process such complex and fluid signaling dynamics to make the all-important decision to infiltrate with a specific trajectory to a marked final destination (Figure 1). The number and nature of the ligands, receptors, and downstream effectors mediating glial infiltration is a largely open question.

Once glia successfully infiltrate to the phagocytosis site, short-distance signals are necessary for the highly specific stage of contact-mediated target recognition (Figure 1). Synapse phagocytosis is a drastic remodeling step, altering information flow through a circuit when connections are eliminated (Vilalta and Brown, 2018; Galloway et al., 2019). Too much or too little glial pruning results in a range of neurological disorders, spanning from early-onset autism spectrum disorder (ASD) to much later schizophrenia in young adults (Chung et al., 2015b; Galloway et al., 2019; Wang et al., 2021). Target recognition involves contact-dependent signals (Figure 1), with highly specific membrane cues on the target synapse binding to their cognate glial receptors (Wilton et al., 2019; Li et al., 2020; Scott-Hewitt et al., 2020). Given the number and density of synaptic connections, these cues need to be highly distinctive, with precise subcellular localization. Following target recognition, glia dramatically rearrange their actin cytoskeleton to engulf the recognized neuronal membrane (Figure 1). Interestingly, multiple pathways appear to be employed for different stages, and also for pruning different parts of the same neuron (Ziegenfuss et al., 2012; Tasdemir-Yilmaz and Freeman, 2014; Vita et al., 2021). For example, mammalian microglia are highly reactive to local signals (De Biase et al., 2017), and upon reception of cues extend pseudopodia projections to invaginate and engulf neuronal material within a phagocytic cup (Figure 1; Barcia et al., 2012). However, very little is known about the signaling mechanisms and signal transduction pathways enabling the formation of these phagocytic cups for any class of glia.

Once the phagocytic cup closes and the material is engulfed, the final step in the glial pruning process is the phagocytosis and digestion of the engulfed material (Figure 1). The newly-formed phagosome is internalized and undergoes a maturation process in which it will fuse with endosomes and lysosomes to ultimately produce a phagolysosome (Desjardins et al., 1994; Sierra et al., 2013). The contents within the phagolysosome will then be broken down and recycled by the glia, completing a phagocytosis event (Figure 1). However, not all synaptic pruning is created equal, as different parts of the synapse are pruned depending on the context. For example, glia can directly phagocytose either the presynaptic bouton, postsynaptic density (PSD) or postsynaptic spine, or whole synapses (Chung et al., 2013; Weinhard et al., 2018; Duffy and Eyo, 2024). This means immediate glial phagocytosis of the target synapse (Figure 1). Alternatively, glia can phagocytose the synaptic ECM (the “synaptomatrix”; Rushton et al., 2020) to induce remodeling indirectly by destabilizing the synapse (Nguyen et al., 2020; Crapser et al., 2021). This results in synapse autophagocytosis and axonal retraction by the neuron (Figure 1). Why would a glia choose to remove the surrounding synaptomatrix as opposed to directly eliminating the synaptic connection? Which signals are involved in guiding the glia in making this decision? These pressing questions remain almost completely unknown. Within the context of this series of steps leading up to glial synaptic pruning (Figure 1), we will now take a closer look at some known molecular mechanisms underlying specific stages, highlighting the many areas where further investigations are needed.

## Directed glial neuropil infiltration

**FIGURE 2:**
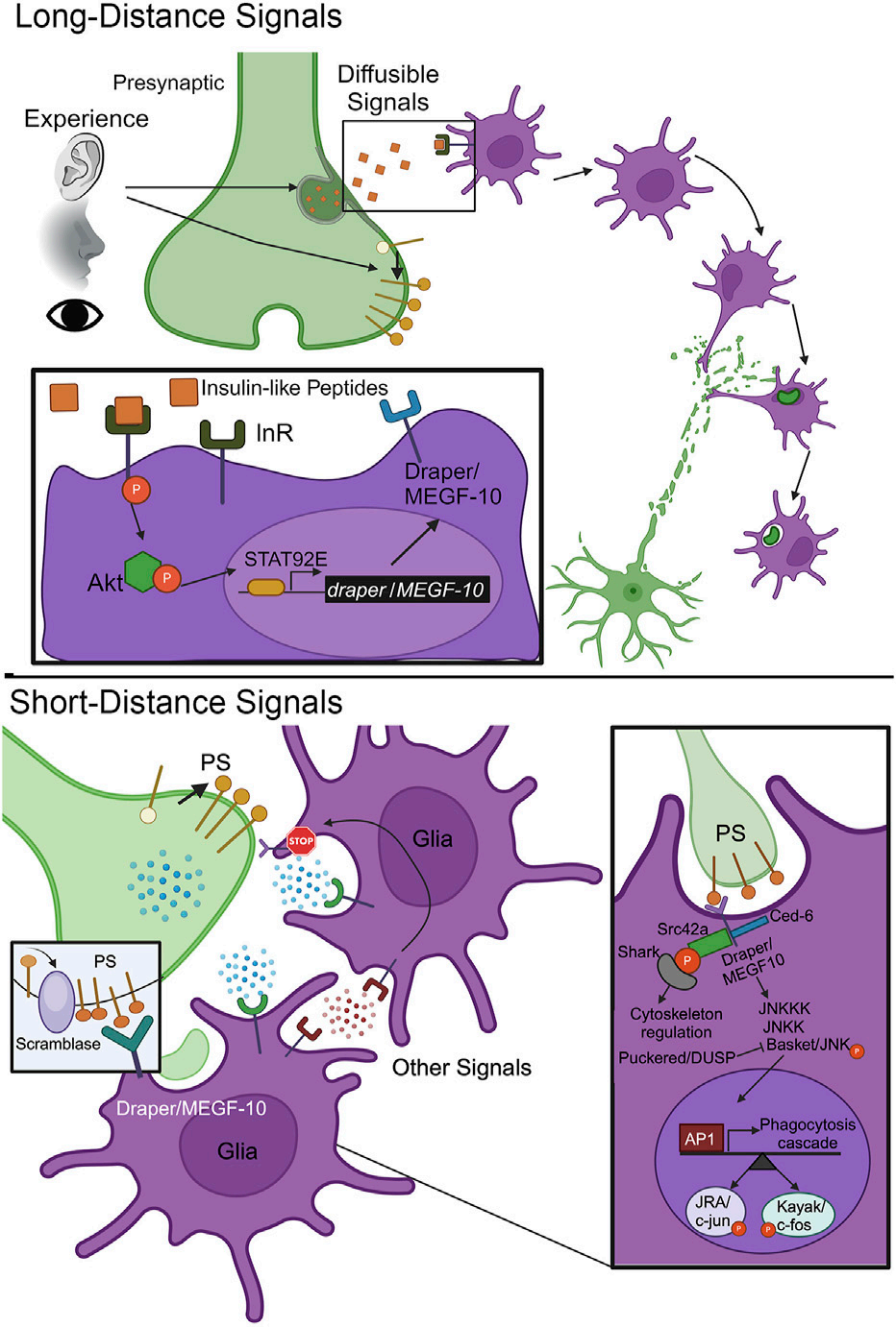
Long-distance and short-distance signals orchestrate synapse pruning. Top, Long Distance Signals: Sensory experience inputs trigger the secretion of diffusible signaling cues that bind cell surface receptors on glia to induce their activation and infiltration to the site of synapse pruning. Inset: Insulin-like peptides (ILPs) bind to the glial insulin receptor (InR) to drive autophosphorylation, downstream Akt1/protein kinase B phosphorylation, and activation of nuclear signal-transducer and activator of transcription protein at 92E (STAT92E). This results in transcriptional upregulation of *draper/MEGF-10* expression to elevate the phagocytosis engulfment receptor in the glial membrane. Bottom, Short Distance Signals: The synapse to be pruned presents cell surface signals to glia receptors to trigger engulfment and phagocytosis. One well-studied cue is the membrane lipid phosphatidylserine (PS), which is flipped from the inner to outer bilayer through the action of a scramblase. Inset: The glial Draper/MEGF-10 receptor binds PS to trigger a signal transduction cascade driving the phosphorylation of Basket/JNK in opposition to Puckered/DUSP phosphatase, phosphorylated Basket/JNK translocation into the glial nucleus, and transcriptional upregulation via Activator Protein-1 (AP-1) JRA/c-Jun and Kayak/c-Fos. In parallel, Draper/MEGF-10 activation phosphorylates Src42a to recruit the SH2 domain kinase Shark and drive cytoskeleton rearrangements for glial engulfment and subsequent phagocytosis.

Underlying the layered stages of glial pruning is the intercellular signaling inducing each step (Wilton et al., 2019; Huo et al., 2024). Mammalian astrocytes and *Drosophila* astrocyte-like glia can form tripartite synapses (Chung et al., 2015a; Semyanov and Verkhratsky, 2021; Saint-Martin and Goda, 2023), so it is possible these glial classes may already be present for synaptic pruning. However, glia are not always in the immediate vicinity, but rather need to be recruited by long-distance signals (Figure 2, top). Direct secretion and extracellular vesicle (EV) signaling are both potential mediators of this intercellular communication (Paolicelli et al., 2019; Ahmad et al., 2022; Ikezu et al., 2024). Mammalian microglia must be recruited (de Deus et al., 2024). Similarly, *Drosophila* astrocyte-like glia are not the predominant phagocytes in either the juvenile or adult brain (Doherty et al., 2009; Freeman, 2015), but rather ensheathing glia acting like microglia mediate synaptic pruning (Baumann et al., 2024; Miller and Broadie, 2024; Nelson et al., 2024b). These glial classes need to be recruited to the site of pruning via long-distance signals (Figure 2, top). Glial infiltration takes time, as experience transformed into neuronal activity needs to generate a maintained signaling gradient, and then glia need time to respond with actin cytoskeleton membrane projections into distant synaptic neuropils (Marmor-Kollet et al., 2023; Nelson et al., 2024b). Synapses are thought to secrete so-called “find me” signals to instruct glial infiltration (Figure 2, top). These signals must diffuse over long distances to instruct glial motility, including activation of transcriptional responses, MMP secretion, and F-actin cytoskeleton regulation instrumental to glial infiltration toward the origin of the signals (Marmor-Kollet et al., 2023; Nelson et al., 2024b). It is important to identify the ligand-receptor interactions mediating such “find me” signaling (Table 1), and the downstream mechanisms of directed glial movement.

**TABLE 1:** List of ligands and receptors involved in the glial phagocytosis of neurons Pathways are separated based on proposed “find me” signaling (top), “eat me” signaling (second from top), “don’t eat me” signaling (second from bottom), and glia-glia signaling (bottom). The columns list ligand, receptor, glial class, stage, model, proposed role, and references. Abbreviations (not defined in the table): Gamma-aminobutyric acid (GABA), 5-hydroxytryptamine (5-HT), interleukin-33 (IL-33), signal regulatory protein α (SIRPα), Mer tyrosine kinase (MERTK), triggering receptor expressed on myeloid cells 2 (TREM2), milk fat globule EGF factor-8 (MFG-E8), six-microns-under (SIMU), olfactory sensory neuron (OSN), mushroom body (MB).

Ligand	Receptor	Glia	Stage	Model	Proposed role	References
“Find me” signaling						
CX_3_CL1 (Fractalkine)	CX_3_CR1	Microglia	Juvenile	Mice	OSN synapse pruning	Gunner et al. (2019)
GABA	GABA_B_R	Microglia	Juvenile	Mice	Inhibitory synapse pruning	Favuzzi et al. (2021)
Insulin-like peptide (ILP)	Insulin Receptor (InR)	Multiple	Juvenile	*Drosophila*	Developmentally transient neuron clearance	Musashe et al., 2016; Song and Broadie, 2023; Vita et al., 2021
Orion	Unknown	Multiple	Development	*Drosophila*	MB axon clearance	Boulanger et al., 2021; Perron et al., 2023
Purines (ATP)	P2RY12	Microglia	Juvenile	Mice	OSN synapse pruning	Sipe et al. (2016)
Spätzle 5 (Spz5)	Toll-6	Cortex	Development	*Drosophila*	Apoptotic neuron clearance	McLaughlin et al. (2019)
“Eat Me” Signaling						
Amyloid precursor protein like (APPL)	Unknown	Multiple	Adult	*Drosophila*	Degenerating axon clearance	Kessissoglou et al., 2020; Song and Broadie, 2023
C1q	CR3	Microglia	Juvenile	Mice	OSN synapse pruning	Schafer et al. (2012)
Phosphatidylserine (PS)	MFG-E8	Microglia	Adult	Mice	Apoptotic neuron clearance	Fuller and Van Eldik (2008)
CD47	SIRPα (Neuronal)	Microglia	Juvenile	Mice	OSN synapse pruning	Jiang et al. (2022)
Pretaporter (Prtp)	Draper/MEGF10/Jedi-1	Multiple	Juvenile	*Drosophila*	Apoptotic neuron clearance	Kuraishi et al., 2009; Song and Broadie, 2023
PS	ADGRG1/GPR56	Microglia	Juvenile	Mice	OSN synapse pruning	Li et al. (2020)
PS	Draper/MEGF10/Jedi-1	Multiple	Development	*Drosophila*	Apoptotic neuron clearance	Ji et al., 2023; Nakano et al., 2019; Tung et al., 2013
PS	MERTK	Microglia	Juvenile	Mice	Inhibitory synapse pruning	Park et al. (2021)
PS	SIMU/Stabilin-2	Multiple	Development	Mice	Apoptotic neuron clearance	Shklyar et al., 2013; Hakim-Mishnaevski et al., 2019
PS	TREM2	Microglia	Juvenile	Mice	OSN synapse pruning	Scott-Hewitt et al. (2020)
“Don’t Eat Me” Signaling						
CD47	SIRPα (Glial)	Microglia	Juvenile	Mice	Prevents OSN synapse pruning	Lehrman et al. (2018)
Neuronal pentraxin-2 (Nptx2)	C1q	Microglia	Adult	Mice	Restrains excitatory synapse pruning	Zhou et al. (2023)
Sushi repeat protein X-linked 2 (SRPX2)	C1q	Microglia	Juvenile	Mice	Prevents OSN synapse pruning	Cong et al. (2020)
Glia-Glia Signaling						
Serotonin (5-HT)	5HT2AR	Unknown	Juvenile	*Drosophila*	Glia signaling synapse pruning	Miller and Broadie (2024)
IL-33	IL1RL1	Multiple	Juvenile	Mice	Glia signaling synapse pruning	Vainchtein et al. (2018)

In *Drosophila*, one “find me” signal may be insulin-like peptides (ILPs; Figure 2, top). *Drosophila* has 8 ILPs, with 7 known to bind the highly conserved insulin receptor (InR; Semaniuk et al., 2021). ILP signaling is known to regulate neurodevelopment, ensuring proper neuron number and connectivity (Rulifson et al., 2002; Luo et al., 2020). In a *Drosophila* injury model, InR signaling via downstream Akt kinase 1 (Akt1; mammalian protein kinase B) effector is required for ensheathing glia clearance of cleaved neuronal axons (Musashe et al., 2016), presumably downstream of ILP release. Glial InR signaling drives the receptor autophosphorylation, Akt1 phosphorylation, and activation of nuclear signal-transducer and activator of transcription protein at 92E (STAT92E; Figure 2, top, inset). The Draper (mammalian MEGF-10) engulfment receptor is thereby upregulated in a STAT92E-dependent mechanism following axotomy (Doherty et al., 2014). Likewise in normal development, glial InR signaling is required for the clearance of developmentally-transient peptide-dispersing factor tritocerebral (PDF-Tri) neurons in *Drosophila* juvenile brains (Vita et al., 2021). Neuronal FMRP drives glial InR activation, with glial-targeted, constitutively-activate InR restoring neuron phagocytic removal in the absence of FMRP (Vita et al., 2021). It is important to investigate ILP-InR signaling in other contexts, such as in experience-dependent critical period synapse pruning (Figure 2, top). Little is known about the molecular cues luring glia to snip connections in an experience-dependent manner, with even less known in *Drosophila*. Future studies are needed to shed light on infiltration signaling, as well as subsequent glial phagocytosis of specific synapses.

## Targeted synapse elimination

Following glial infiltration, synapse-selective, contact-dependent cues control the precise pruning of target synapses (Figure 2, bottom). These “eat me” signals trigger glial engulfment and phagocytosis (Wilton et al., 2019; Nagappan-Chettiar et al., 2023). There is evidence for both direct glial synapse engulfment and indirect ECM/PSD phagocytosis driving synapse pruning (Weinhard et al., 2018; Nguyen et al., 2020; Crapser et al., 2021). One of the best-established “eat me” signals is phosphatidylserine (PS; Segawa and Nagata, 2015; Scott-Hewitt et al., 2020; Nagappan-Chettiar et al., 2023). PS is normally found in the plasma membrane inner leaflet, but flips to the outside leaflet via the action of a scramblase (Figure 2, bottom), to serve as a phagocytic ligand (Naeini et al., 2020). PS serves as a ligand for a variety of mammalian glial receptors (Table 1), which opens the idea of decision-making at the receptor level, with the potential for sequential, competitive, and synergistic interactions (Ziegenfuss et al., 2012; Tasdemir-Yilmaz and Freeman, 2014). For example, G protein-coupled receptor 56 (GPR56) binds PS for microglial visual retinogeniculate synapse pruning in juvenile mice (Li et al., 2020). However, triggering receptor expressed on myeloid cells 2 (TREM2) is necessary for refining both hippocampal and retinogeniculate synapses in juvenile mice (Table 1), which fails to occur when PS exposure is disrupted (Scott-Hewitt et al., 2020). Moreover, Mer tyrosine kinase (MERTK) receptors bind PS in inhibitory synapses to signal microglial pruning in adult mice (Park et al., 2021). The PS “eat me” signal and conserved receptors similarly function in *Drosophila*, with this forward genetic model providing a powerful avenue to investigate signaling mechanisms and downstream signal transduction.

In *Drosophila* dendritic arborization (da) neurons, PS exposure drives pruning both during normal development and following injury (Sapar et al., 2018). Externalization can be ectopically driven by disrupting flippase control of PS asymmetry with scramblase overexpression (Figure 2, bottom). Draper (mammalian MEGF-10) receptors are proposed to bind PS in injury models (Tung et al., 2013; Sapar et al., 2018; Ji et al., 2023) to mediate neuronal clearance (Doherty et al., 2009; Lu et al., 2017). Draper/MEGF-10 activation induces phosphorylation of Basket (mammalian JNK), negatively regulated by Puckered (mammalian DUSP) phosphatase, driving translocation into the glial nucleus (Figure 2, bottom, inset). This signaling induces the Activator Protein-1 (AP-1) transcriptional regulators, Jun-related antigen (Jra; mammalian c-Jun) and Kayak (mammalian c-Fos), elevating AP-1-dependent genes to facilitate glial pruning (MacDonald et al., 2013; Purice et al., 2017). Genes include secreted synaptomatrix remodeler MMP-1 (Dear et al., 2016; 2017) and F-actin scaffold Cheerio (mammalian FLNA; Külshammer and Uhlirova, 2013). Draper/MEGF-10 activation also phosphorylates Src42a to recruit SH2 domain kinase Shark and drive cytoskeleton rearrangement for glial phagocytosis (Figure 2, bottom, inset; Freeman, 2015). Interestingly, the chemokine Orion has been shown to bridge PS to Draper/MEGF-10 during glial phagocytosis (Table 1), acting as one of several known secreted PS adaptors (Ji et al., 2023). These studies provide a detailed framework of PS- Draper/MEGF-10 signaling mechanisms in the glial phagocytosis injury context. However, roles in normal juvenile brain development remain much less characterized.

In the *Drosophila* juvenile brain, glial phagocytosis of the transient PDF-Tri neurons similarly requires the Draper/MEGF-10 engulfment receptor (Vita et al., 2021). The FMRP translational regulator positively regulates glial Draper levels in this mechanism. Likewise in the olfactory critical period, ensheathing glia require the Draper/MEGF-10 receptor for experience-dependent synapse pruning (Nelson et al., 2024b). This signaling mediates phosphorylated Basket/JNK nuclear translocation in glia, as above (Figure 2, bottom, inset), to upregulate Cheerio/FLNA and thus control experience-dependent actin cytoskeleton dynamics. Acting as an F-actin crosslinking signaling scaffold, Cheerio/FLNA is required for the experience-dependent infiltration phagocytosis of synapses (Nelson et al., 2024b). In parallel, critical period experience drives extracellular signal-regulated kinase (ERK) signaling in glia in a dose-dependent mechanism (Baumann et al., 2024). Such glial ERK signaling drives normal synaptic pruning, and a Noonan Syndrome patient-derived protein tyrosine phosphatase non-receptor type 11 (PTPN11) mutation within glia increases ERK signaling to exacerbate experience-dependent synaptic pruning (Baumann et al., 2024). It is imperative to determine whether the PS “eat me” signal is also utilized as a localized ligand for the Draper/MEGF-10 receptor in experience-dependent synaptic pruning in the olfactory critical period. If so, we predict that temporally-restricted odorant experience can activate a synaptic scramblase to expose PS on target synapses and thus signal specific glial phagocytosis. It will be essential to place such signaling in the context of other ligand-receptor interactions mediating the timing and specificity of glial synapse pruning.

## Neuron-to-glia signaling mechanisms

An expanding array of ligands and receptors involved in glial phagocytosis have been identified in mammals and *Drosophila*, categorized into “find me,” “eat me,” and “don’t eat me” pathways (Table 1). In mammalian visual cortex ocular dominance plasticity (ODP), microglia respond to monocular deprivation by changing morphology to infiltrate and then engulf synapses in an experience-dependent mechanism (Schafer et al., 2012; Sipe et al., 2016), although a recent study reports microglia are dispensable for this critical period synapse remodeling (Brown et al., 2024). Mice lacking P2RY12 purinergic receptors reportedly lack synaptic pruning (Sipe et al., 2016), although the CS1R inhibitor PLX5622 introduced at P14, when experience-dependent visual cortex begins, reportedly does not block this process (Brown et al., 2024). As a “find me” signal, neuronally-released ATP is proposed to be required for microglial P2YR12 reception and synaptic pruning (Table 1). During cortical development, specialized microglia receptive to GABA are proposed to prune inhibitory synapses (Favuzzi et al., 2021). GABA reception induces transcriptional changes within these primed microglia, leading to preferential pruning of cortical inhibitory synapses during a mouse critical period (Table 1). In the rodent whisker barrel cortex, microglia have been shown to respond to the reduction in neural activity following removal of whiskers (Gunner et al., 2019). Following sensory lesioning, microglia engulf and eliminate thalamocortical synapses in layer IV of the barrel cortex. This synapse pruning requires CX_3_CR1 (Table 1), the microglial receptor for neuronal release of the chemokine fractalkine (CX_3_CL1) (Hoshiko et al., 2012; Gunner et al., 2019).

Microglial CX_3_CR1 is also required for infiltration and pruning of dendritic spines in the hippocampus (Paolicelli et al., 2011). Depletion of CX_3_CR1 in mice reduces microglial density in the hippocampus, suggesting soluble release of fractalkine promotes microglial infiltration for subsequent synapse refinement during development (Paolicelli et al., 2011). Similarly in *Drosophila*, neuronal chemokine Orion induces astrocyte-like glial infiltration into the mushroom body (MB) and subsequent pruning of MB γ axons (Boulanger et al., 2021). Orion shares similarities to mammalian fractalkine, such as the CX_3_C motif, and plays a role in transforming/activating astrocytes into phagocytes (Perron et al., 2023). Orion also plays a role in multiple additional developmental pruning contexts, including clearance of transient PDF-Tri and vCorazonin^+^ (vCrz^+^) neurons (Table 1). Considering the shared glial pruning mechanisms (Tasdemir-Yilmaz and Freeman, 2014; Vita et al., 2021; Song and Broadie, 2023), it is surprising that Orion is dispensable for olfactory sensory neuron (OSN) clearance following axotomy (Perron et al., 2023). *Drosophila* utilizes another “find me” signal to induce apoptotic neuron clearance by instead tapping into the innate immune system. Dying neurons in a *Drosophila* larval brain lobe release soluble Spätzle 5 (Spz5; Table 1) to activate and recruit cortex glia for their phagocytosis (McLaughlin et al., 2019). Glial Toll-6 receptors bind Spz5, initiating a signal transduction cascade resulting in FoxO-dependent transcription and upregulation of Draper/MEGF-10 for neuronal clearance phagocytosis. Note this is not the first case of glia utilizing innate immune system signals, as microglia use them as downstream “eat me” signals.

Following “find me” signals, “eat me” signals directly induce glial engulfment and subsequent phagocytosis (Table 1). During development, it was first proposed that C1q, an initiator of the classical complement cascade, is expressed and localized to synapses throughout the CNS and retina in postnatal mice (Stevens et al., 2007). Later work demonstrated that microglia express complement receptor 3 (CR3; Table 1), which binds C1q to drive microglial engulfment and phagocytosis of retinogeniculate synapses in an activity-dependent mechanism (Schafer et al., 2012). Interestingly, in the developing mouse retina, neuronal signal regulatory protein alpha (SIRPα; Table 1) acts as a permissive cue for developmental microglia-mediated phagocytosis (Jiang et al., 2022). Microglia also express SIRPα and SIRPα-CD47 cascades to inhibit pruning (discussed below), but neuronal expression of SIRPα reportedly binds neuronal CD47 to modulate the inhibitory signal accessibility, thereby promoting glial synapse pruning (Jiang et al., 2022). Microglia have also been suggested to recognize and bind neuronal PS with secreted milk fat globule EGF factor-8 (MFG-E8) as an adaptor for apoptotic neurons (Fuller and Van Eldik, 2008). In *Drosophila*, Draper/MEGF-10 is also suggested to bind PS (Tung et al., 2013), and Pretaporter (Prtp; Table 1) is proposed to be an additional Draper/MEGF-10 ligand (Kuraishi et al., 2009; Nakano et al., 2019). Neuronal Prtp signals glial Draper/MEGF-10-mediated clearance of developmentally-transient PDF-Tri neurons (Song and Broadie, 2023). The neuronal amyloid precursor protein like (APPL) signal (Table 1) also drives glial phagocytosis of these apoptotic neurons (Kessissoglou et al., 2020; Song and Broadie, 2023).

During *Drosophila* embryogenesis, glia utilize both Draper/MEGF-10 and Six-Microns-Under (SIMU; mammalian Stabilin-2) receptors to phagocytose developmentally apoptotic neurons (Kurant et al., 2008; Shklyar et al., 2013; Hilu-Dadia et al., 2025). Similarly, larval vCrz^+^ neurons are cleared by astrocyte-like glia during metamorphosis with Draper/MEGF-10 required for the neuronal cell bodies and the Myoblast City (Mbc)/Crk/dCed-12 complex for the neuronal processes (Shklyar et al., 2014; Tasdemir-Yilmaz and Freeman, 2014; Hilu-Dadia et al., 2018). In *Drosophila* injury models, this later pathway is used sequentially from glial activation through engulfment, internalization, and degradation (Ziegenfuss et al., 2012). Moreover, downstream of receptor kinase (DRK; mammalian Grb2), the Daughter of Sevenless (DOS; mammalian Gab2) adapter protein and Son of Sevenless (SOS; mammalian mSOS) guanine nucleotide exchange factor (GEF) are required for glial activation through subsequent engulfment and degradation following axotomy (Lu et al., 2014). Downstream of the Draper/MEGF-10 receptor, the DRK/DOS/SOS and Mbc/Crk/dCed-12 complexes converge to activate the Rac1 GTPase for efficient glial phagocytosis in response to neural injury (Lu et al., 2014). Similarly, disruption of the trimeric protein phosphatase 4 (PP4) serine/threonine phosphatase complex impairs glial recruitment to neural injury sites and delays clearance (Winfree et al., 2017). Downstream of the Draper/MEGF-10 receptor, PP4 is molecularly coupled to Rac1 via the SOS GEF complex, suggesting involvement in glial actin cytoskeleton remodeling to enable glial infiltration phagocytosis following axotomy injury (Winfree et al., 2017).

Beyond “find me” and “eat me” signals, there are also inhibitory “don’t eat me” signals preventing glial phagocytosis (Table 1). These local signals present on synapses counter the wide array of positive signals, such as PS or complement C1q tagging of synapses (Schafer et al., 2012; Scott-Hewitt et al., 2020). The best known “don’t eat me” pathway is neuronal CD47 binding to microglial receptor SIRPα (Lehrman et al., 2018; Jiang et al., 2022). CD47 helps microglial activity-dependent discrimination in engulfment, with CD47 deficient mice exhibiting elevated pruning, with less active inputs engulfed less often than normal (Lehrman et al., 2018). Interestingly, SIRPα serves a dual purpose to prohibit synapse pruning (Jiang et al., 2022). It would be fascinating to investigate the mechanisms by which a synapse decides which role SIRPα serves (Table 1). Neuronal pentraxin-2 (Nptx2) also acts as a “don’t eat me” signal by binding C1q, to inhibit microglial synaptic pruning of excitatory cortical neurons in adult mice (Zhou et al., 2023). Likewise, sushi repeat protein X-linked 2 (SRPX2; Table 1) also binds C1q to block signaling activity, thus minimizing microglial complement-dependent retinogeniculate synapse elimination in juvenile mice (Cong et al., 2020). While both “don’t eat me” signals function through the C1q receptor, SRPX2 only does so during development, whereas Nptx2 operates in adult brain synapse pruning in mice. However, as far as we are aware, there have not yet been identified “don’t eat me” signals for *Drosophila* glia phagocytosis synaptic pruning. While much focus is understandably on neuron-to-glia signaling, glia-to-glia signals have recently emerged with essential roles in experience-dependent synapse pruning.

## Glia-to-glia signaling mechanisms

A much less investigated question is whether glia communicate with each other to promote or inhibit synapse pruning (Table 1, bottom). Mammalian astrocytes have been shown to release interleukin-33 (IL-33), which is then received by microglia IL1RL1 receptors to positively induce microglial synapse engulfment and phagocytosis in the spinal cord and thalamus of juvenile mice (Vainchtein et al., 2018). In the *Drosophila* olfactory critical period, glial-glial communication was also recently shown to be essential for experience-dependent synapse pruning (Miller and Broadie, 2024). Surprisingly, both glial serotonin (5-HT) production and reception are required for critical period synapse remodeling (Table 1, bottom). Early-life olfactory experience elevates dose-dependent serotonin production within glia, but no change in the mature brain, demonstrating a temporally-restricted mechanism in the juvenile critical period (Miller and Broadie, 2024). Glia also express the 5-HT_2A_ receptor, which is likewise induced only by critical period experience and totally required for critical period experience-dependent synapse pruning (Table 1, bottom). The glial 5-HT_2A_ receptor is rate-limiting for synapse elimination in response to odorant experience during the critical period (Miller and Broadie, 2024). Strikingly, conditional glial over-expression of either the biosynthesis enzyme tryptophan hydroxylase (Trhn) and 5-HT_2A_ receptors in adults, well past the normal critical period, enables *de novo* experience-dependent synapse pruning, thus re-opening critical period-like plasticity (Miller and Broadie, 2024). Future studies are needed to determine how and why glia-to-glia serotonin signaling is essential for critical period experience-dependent synapse pruning, reveal the specific glial classes involved in this novel mechanism, and test this potent means of re-opening critical period synapse pruning capabilities.

## Challenges and future directions

Critical period experience-dependent synapse pruning may occur via either glial phagocytosis or neuron cell-intrinsic mechanisms, such as ubiquitin-proteasome system (UPS) activation (Watts et al., 2003; Kuo et al., 2005) coupled to lysosome-dependent autophagocytosis (Birdsall and Waites, 2019). Indeed, these options may be linked by glial phagocytosis of the synaptic ECM (“synaptomatrix”; Rushton et al., 2020), which can undermine synapse stability and thus cause later synapse retraction (Nguyen et al., 2020; Crapser et al., 2021). It will be vital to investigate this hypothesis in both mammalian and *Drosophila* models. A constant challenge in exploring glial function is imaging limitations owing to the spatiotemporal scale of synapse pruning. It is often difficult to convincingly ascertain glial phagocytosis mechanisms (Andoh and Koyama, 2021). Moreover, most studies use fixed brain imaging, which offers a single snapshot of the process where it is difficult to interpret the dynamic cascade of glial activation, infiltration, target recognition, engulfment, and phagocytosis (Figure 1). This makes it especially challenging to tease apart the signaling pathways involved at each stage of synaptic pruning (Morini et al., 2021). For example, if a glial gene involved in phagocytosis is removed, and a snapshot captures both lack of glial infiltration and pruning, one is likely to conclude the gene is required for only the first step. However, did the glia fail to infiltrate, or did glia infiltrate, not have the correct “eat me” signals, and thus retract as a result? A snapshot is likely inadequate to separate these possibilities. This conundrum is worsened when we broaden the synaptic pruning decision-making cascade beyond just “find me” and “eat me” signals (Figure 1).

An additional challenge is understanding the different timescales of “find me” and “eat me” signaling (Figure 2). It takes time for synapses to release signals following critical period experience, and more time for such signals to diffuse, reach distant glia, establish a stable gradient, initiate activation, and finally for glia to directionally infiltrate the neuropil and prune synapses (Yousefpour et al., 2023). Thus, “find me” signaling is slow and needs to be sustained. Future studies are needed to link experience to the production of such signals, and to test the hypothesis of gradient-directed glial infiltration. Glial phagocytosis based on “eat me” signaling presumably takes place on a much more rapid timescale. In *Drosophila* injury models, clearance of axons starts a day after axotomy, with near full removal requiring 5 days (MacDonald et al., 2006; Doherty et al., 2009; Ziegenfuss et al., 2012). Likewise, the normal clearance of developmentally transient PDF-Tri neurons from the *Drosophila* juvenile brain similarly starts on the day following eclosion, with full removal requiring 5 days (Vita et al., 2021; Song and Broadie, 2023). Interestingly, overlapping olfactory experience-dependent critical period synaptic pruning occurs over just 2 days (Golovin et al., 2019; Golovin et al., 2021), with recent studies demonstrating that the vast majority of the synapse elimination occurs within the first 24 h of the critical period (Baumann et al., 2024; Miller and Broadie, 2024; Nelson et al., 2024a; Nelson et al., 2024b). Both injury and experience-dependent critical period models assay olfactory sensory axon clearance or synapse pruning, so what determines the relative phagocytosis timing? This timescale conundrum needs to address the role of temporally-restricted experience input, glial activation/infiltration, and subsequent “eat me” signaling for phagocytosis in a much later timeframe.

 In addition to timing constraints, the tagging of targeted synapses for glial pruning requires locally retained signals (e.g., extracellular ILP gradients (long-term recruitment), PS maintenance in the liquid membrane (synapse specificity); Figure 2). Complicating the establishment of this signaling hierarchy is the fact that signals are not static. Secreted “find me” signals must traverse the complex ECM and presumably must do so in a manner that generates a gradient sufficient for directed glial infiltration (Ravichandran, 2010). Likewise, local “eat me” and “don’t eat me” signals in synaptic membranes are subject to the dynamic mosaic properties of the lipid bilayer (Nicolson and Ferreira de Mattos, 2023), where rapid diffusion occurs unless cues are securely anchored (Jacobson et al., 2019). How are phospholipids, for example, kept at a single synapse? How many signaling molecules are sufficient to illicit pruning (1, 10, 100, more)? Future studies exploring these questions are needed. Complicating this question is the occurrence of multiple receptors for a single ligand (e.g., PS; Table 1), as well as multiple ligands binding the same receptor (e.g., Draper/MEGF-10; Table 1). Is this a simple case of the same molecular machinery being co-opted in different pruning contexts (Lu et al., 2017; Nelson et al., 2024b), or does it open the door to sequential, competitive, or synergistic signaling mechanisms in a single synapse pruning event (Ziegenfuss et al., 2012; Tasdemir-Yilmaz and Freeman, 2014)? How do glia integrate signals to make the critical decision to infiltrate/prune? In the case of Draper/MEGF-10, is this decision being made with a single receptor binding multiple ligands (Table 1) for different mechanistic stages? Future work is needed to address the cavalcade of ligands and receptors interacting in the glial pruning signaling hierarchy, as well as the potential interplay and overlap of these signaling levels.

The idea of glial ECM phagocytosis to drive synapse retraction rather than direct glial engulfment of synapses is intriguing (Nguyen et al., 2020; Crapser et al., 2021). Synaptomatrix deposition has long been proposed as a core aspect of critical period closure (Wang and Fawcett, 2012; Willis et al., 2022), perhaps because it is challenging for glia to infiltrate and mediate synapse pruning (Purice et al., 2017; Crapser et al., 2021). In addition, the synaptomatrix has long been proposed to stabilize synaptic connections (Yang et al., 2023), and compromising this specialized ECM environment can destabilize synapses and cause their rapid retraction (Nguyen et al., 2020; Crapser et al., 2021). Microglia are implicated in ECM modulation (Crapser et al., 2021). In mice, hippocampal neurons release cytokine IL-33 which instructs microglia to engulf and degrade the ECM (Table 1; Nguyen et al., 2020). Thus, glia may alter the synaptomatrix foundation to locally destabilize synapses, resulting in subsequent synapse retraction via neuron cell-intrinsic autophagocytosis (Birdsall and Waites, 2019). How is this regulated, also considering glial MMP functions? Astrocyte regulation of ECM composition has already been shown to precociously close the visual critical period in mice (Ribot et al., 2021), further highlighting astrocytic roles in closing critical periods of heightened plasticity (Ackerman et al., 2021; Lloret-Fernández et al., 2023). The *Drosophila* genetic system provides a particularly powerful toolkit to manipulate the glycosylated synaptomatrix (Dani and Broadie, 2012; Jumbo-Lucioni et al., 2014) as well as a reduced MMP proteome (Dear et al., 2016). This model thus presents a promising research avenue, particularly with the new olfactory critical period (Baumann et al., 2024; Miller and Broadie, 2024; Nelson et al., 2024b).

The role of direct glia-glia signaling interactions in experience-dependent synapse pruning offers an exciting new level of regulatory mechanism (Vainchtein et al., 2018). For example, glia-glia serotonin signaling was recently shown to be absolutely essential for the *Drosophila* olfactory critical period (Miller and Broadie, 2024), but it has yet to be determined if this represents glial autocrine signaling or an interaction between different glial classes. Overall, the mechanistic details of this glia-glia serotonin signaling still need to be explored. Both mammalian and *Drosophila* glial classes have distinct pruning roles in a wide variety of contexts (Allen and Lyons, 2018; Vainchtein et al., 2018). Interestingly, chemokine-like Orion (Table 1) acts on all three glial phagocytes in the *Drosophila* brain (cortex, ensheathing, and astrocyte-like glia; Boulanger et al., 2021; Ji et al., 2023; Perron et al., 2023), raising the question of how Orion directs specific glial pruning. Cortex and ensheathing glia combinatorically remove PDF-Tri neurons within the young juvenile brain (Vita et al., 2021; Perron et al., 2023; Song and Broadie, 2023), ensheathing glia alone clear OSN axons following injury (Doherty et al., 2009) and in experience-dependent critical period synapse pruning (Baumann et al., 2024; Miller and Broadie, 2024; Nelson et al., 2024b), and astrocyte-like glia remove MB γ axons during development (Tasdemir-Yilmaz and Freeman, 2014). How is this glial class coordination orchestrated? This question becomes more challenging considering that many pruning events happen at the same time, including juvenile brain removal of the developmentally-transient PDF-Tri neurons (Vita et al., 2021; Song and Broadie, 2023) and experience-dependent OSN synapse pruning (Miller and Broadie, 2024; Nelson et al., 2024b). Although these events occur in separate neuropils, they are closely adjacent in the early-life juvenile brain.

Re-opening critical period-like remodeling capabilities at maturity has become a beacon for the potential treatment of trauma, acute injury, and inherited disease states (Hensch and Bilimoria, 2012; Nardou et al., 2023). We have already implicated glial circuit pruning dysfunction in *Drosophila* models of Fragile X syndrome and Noonan syndrome (Vita et al., 2021; Song and Broadie, 2023; Baumann et al., 2024). Considering that glial synaptic pruning dysfunction is linked to multiple disorders, reopening critical periods or releasing the brakes that limit glia-mediated synaptic pruning in the adult brain could be a promising way to treat these conditions. Future studies exploring how critical period synaptic pruning impacts sensory processing and learning/memory abilities are a high priority, with the re-opening of critical periods potentially offering an attractive therapeutic avenue. Moreover, inducing experience-dependent glial pruning activities at maturity could be important in a range of other neurological disorders linked to glial dysfunction (Starkey et al., 2023). In this pursuit, conditional neuronal ILP signaling or the conditional constitutive activation of glial insulin receptors could possibly induce post-critical period remodeling capabilities (Figure 2, top). Likewise, the conditional neuronal induction of PS exposure with scramblases, secreted PS signaling adaptors, or glial Draper/MEGF-10 receptors might also drive adult brain experience-dependent synapse remodeling (Figure 2, bottom). We have already discovered that the conditional induction of glial serotonin signaling or 5-HT_2A_ receptors within the mature adult brain can re-open experience-dependent glial synapse pruning (Miller and Broadie, 2024), to produce circuit plasticity properties indistinguishable from the juvenile critical period. Future studies are needed to test glia-to-glia class signaling in this context, and to dissect the mechanistic requirements of glial serotonergic signaling for synapse pruning within the overall context of glial activation, “find me” infiltration, and “eat me” phagocytosis.
